# Scorpion Toxins Specific for Potassium (K^+^) Channels: A Historical Overview of Peptide Bioengineering

**DOI:** 10.3390/toxins4111082

**Published:** 2012-11-01

**Authors:** Zachary L. Bergeron, Jon-Paul Bingham

**Affiliations:** Department of Molecular Biosciences and Bioengineering, College of Tropical Agriculture and Human Resources, University of Hawaii at Manoa, Honolulu, HI 96822, USA; Email: zacharyb@hawaii.edu

**Keywords:** scorpion, peptide, toxin, potassium (K^+^) channel, bioengineering, probe, chimera, cyclotide, molecular therapeutic

## Abstract

Scorpion toxins have been central to the investigation and understanding of the physiological role of potassium (K^+^) channels and their expansive function in membrane biophysics. As highly specific probes, toxins have revealed a great deal about channel structure and the correlation between mutations, altered regulation and a number of human pathologies. Radio- and fluorescently-labeled toxin isoforms have contributed to localization studies of channel subtypes in expressing cells, and have been further used in competitive displacement assays for the identification of additional novel ligands for use in research and medicine. Chimeric toxins have been designed from multiple peptide scaffolds to probe channel isoform specificity, while advanced epitope chimerization has aided in the development of novel molecular therapeutics. Peptide backbone cyclization has been utilized to enhance therapeutic efficiency by augmenting serum stability and toxin half-life *in vivo* as a number of K^+^-channel isoforms have been identified with essential roles in disease states ranging from HIV, T-cell mediated autoimmune disease and hypertension to various cardiac arrhythmias and Malaria. Bioengineered scorpion toxins have been monumental to the evolution of channel science, and are now serving as templates for the development of invaluable experimental molecular therapeutics.

## 1. Introduction

Scorpion toxins represent a vast bio-cache (~100,000) of pharmacologically relevant peptide-toxins that have provided an important foundation for advancing the study and understanding of various sodium (Na^+^), potassium (K^+^), chloride (Cl^−^) and calcium (Ca^2+^) ion channels, and their associated pathologies [1]. Of these, K^+^ channels (KCN), have shown promise as potential therapeutic targets for the treatment of a myriad of human diseases ranging from asthma [2] diabetes, angina, cardiac ischemia and hypertension [3] to chronic inflammation, autoimmune disease and cancer [4]. 

From their preliminary use as ligands to study the basis and selectivity of ion-channel electrophysiology, scorpion toxins have steadily evolved into a versatile bioengineering platform for designing functional probes, with a diverse range of applications including the localization of ion channels in cellular models [5], purification of novel ion-channels [6], discerning isoform selectivity [7], serving as molecular therapeutics [8], and even advancing visualization of tumor foci for improved detection and intraoperative resection of life threatening malignancies [9]. These efforts are leading to an increased value in their therapeutic development and application, which serves to augment and diversify our present understanding of ion-channel pathophysiologies.

This review focuses on the process of scorpion toxin bioengineering and illustrates key developments in their evolution and application as KCN probes. To do this we provide a basic overview of scorpion biology, illustrate the use of scorpion toxins to investigate KCN; then advance to the study of peptide-toxin structure and its importance in establishing structure activity relationships, finally illustrating the transition via bioengineering to advance scorpion toxin analogues as biopharmaceuticals and therapeutic tools.

## 2. Scorpion Biology

There are approximately 1500 different species of scorpion. Most of which toxinologically represent little danger to humans [10]. There are however several species (~25) which are known to be capable of causing human fatalities [11], with the majority of these belonging to the “old world” family Buthidae, widely distributed in the afrotropical and palaearctic ecozones [12]. The most poignant example of which is the Indian Red Scorpion (*Hottentotta tamulus*), generally recognized as the most lethal of all scorpion species [13].

**Figure 1:**
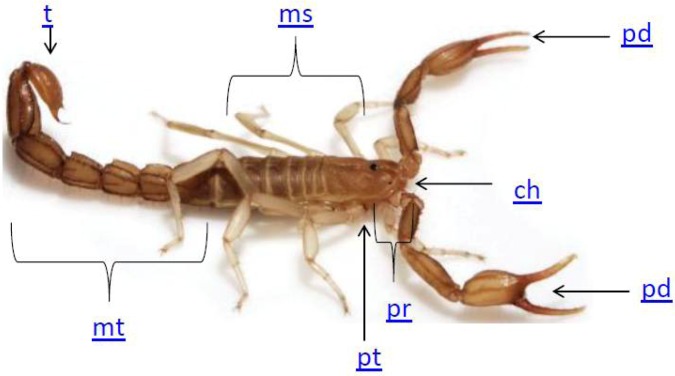
Basic Scorpion morphology. The body is divided into three major sections, the tail or metasome (**mt**); the abdomen or mesosoma (**ms**); and the head region or prosoma (**pr**); Distinct structures are also highlighted including pinchers or pedipalp (**pd**); mandibles or chelicerae (**ch**); contact chemosensors (pectines-pt), and the venom apperatus or telson (**t**). Adapted from Weber *et al*. 2012 [15].

Physiologically, scorpions display characteristic features, but have changed little over the millennia. Appearing in the fossil record nearly 450 million years ago during the middle Silurian period, scorpions are segmented animals displaying fundamental physiology including a head (prosoma), abdomen (mesosoma) and tail (metasoma). Major appendages include chelate pedipalps (pinchers), chelicerae (morphologically related to mandibles), pectines (contact chemosensors), eight legs (arranged in four sets of two), and a telson (for venom delivery in predation and defense) at the apex of the tail [14]; see Figure 1. 

The geographic distribution of scorpions is immense, with known colonization of every landmass short of Antarctica. Acclimatization to all non-boreal habitats has occurred including desert, savannah, grasslands, temperate forests, tropical forests, rain forests and intertidal zones; from as high as 5500 m in elevation, to 800 m below ground [16]. Ecologically speaking, in many habitats scorpions have become fundamental in terms of predation, density, diversity and standing biomass [11]. 

Scorpions are the athletes of the arachnid world, dexterous in defense and prey capture. Known to be voracious eaters, their diet includes insects, spiders and other scorpions, extending to snakes, lizards and even rodents. Amazingly, even flying insects are little match for the lightning quick pedipalps of these predators. In order to determine the strategy required for successful prey capture, scorpions are capable of analyzing the predator to prey size ratio, and evaluate how one should engage in mortal combat. Generally, small prey is crushed with the pedipalps, while larger or unsecured prey receives a sting and subsequent envenomation. Adult scorpions and those species with large pedipalps tend to crush their prey, while smaller scorpions and those with small pedipalps, sting and utilize venom in order to subdue, which may require multiple injections. Prey is often oriented head first and consumed. Interestingly arachnids, including scorpions, are thought to be the first to implement the use of toxins in prey capture/defense [17] indicating a high degree of evolution resulting in toxin-receptor isoform selectivity. 

Upon prey impalement, the scorpion injects a venom comprised of phyla specific neurotoxins (toxins), which are known to cause up to 5000 human fatalities a year [1,18]. Initially, with the isolation of Scorpamins in 1961 [19], the resulting biologically active toxins were thought to be multimeric proteins. In 1967, Rochat *et al*. found that the bioactive toxins were actually composed of single polypeptide chains, 63 to 64 amino acids (AAs) in length, held in a specific three-dimensional conformation through internal-disulfide bridging [20]. Subsequent analysis has revealed the complexity of the venom which consists of a cocktail of low molecular weight proteins, oligopeptides, free AAs, nucleotides, low molecular weight salts and organic compounds [21]. 

## 3. The Use of Scorpion Toxins to Investigate Potassium Channels

The foundation for our current knowledge regarding the pharmacological action of scorpion toxins was generated mainly through electrophysiological experiments on isolated muscle and nerve cells using voltage clamping (or later, patch-clamp). Inspired by the work of Cole in 1949 [22], Hodgkin and Huxley first described ground breaking experiments, for which they would later win the Nobel Prize, whereby ionic current (in the form of resistance) was measured across the membrane of an excitable cell or tissue type, in their case, giant squid axon (*Loligo vulgaris*) [23]. This foundational work now provides us the basis of modern electrophysiology, where we now understand that ion channels control the flux of ions that cause the generation and propagation of nerve impulses and action potentials. Molecular dissection of ion channels using scorpion peptide toxins, has lead to direct evidence that specific mutations, phenotypically expressed as channel up/down regulation, can be correlated to changes, sometimes fatal, in the propagation and conduction of electrical impulses in the body. As evidenced by sudden death heart attacks manifested by underlying Long QT Syndrome, a channelopathy associated with the regulation and expression of the potassium channel K_V_11.1 from the human Ether-a-go-go Related Gene (hERG) [24]. 

The first reported interaction between scorpion venom and KCNs was in 1982, by Carbone *et al*. when venom preparation obtained from the Mexican scorpion *Centruroides noxius* was applied to a giant squid axon, while monitoring by means of voltage-clamp [25]. The single constituent responsible for the observed activity was Noxiustoxin (NTX), a 39 AA peptide purified from homogenized crude venom extract, and separated by Sephadex G-50 chromatography, followed by ion-exchange [26]. Although isolated and purified, the multi-faceted potential of the toxin was not yet realized. Over the next few years, several additional scorpion toxins were reported with distinct KCN activity. With advances in chromatographic technology, combined with the establishment of single channel recordings, the use of this new class of toxins was expanded in 1985, when Miller *et al*. first used Charybdotoxin (*Leiurus quinquestriatus*; ChTx) to identify and pharmacologically characterize a novel Ca^2+^ regulated KCN, now known as K_Ca_1.1, MaxiK or BK [6,27,28]. 

As the decade progressed, the number of selective peptide toxins increased. These receptor specific research probe discoveries were augmented by scorpion diversity, intraspecial variation and the introduction of novel purification techniques such as Reverse Phase-High Performance liquid chromatography (RP-HPLC) [29]. The high-pressure approach had many advantages over traditional gravity based (low pressure) separation technologies including increased limits of detection and purity [27]. The primary approach to defining and characterizing purified peptides at the time was by *N*-terminal Edman degradation, which had various limitations such as lack of recognition for most post-translational modifications (PTMs) [30] which are now known to functionally augment potency and isoform selectivity of scorpion toxins. 

The expanded number of ion channel probes combined with an advanced understanding of toxin-receptor interactions, facilitated the development of a technique known as pore mapping [31,32] or molecular footprinting [33], which stimulated elucidation of structural determinants in isoform selectivity. Also used as immunogens, toxins can be utilized to produce site-directed anti-peptide antibodies. These in turn can neutralize the toxin-induced lethal effects *in vivo*—an imperative aspect of anti-scorpionic serotherapy in the treatment of human envenomation. The expansion of scorpion toxin discovery represents the establishment of a novel set of tools to study medically significant KCN isoforms, and further, has stimulated the incorporation of receptor physiology in disease models [34]. 

## 4. Peptide-Toxin Structure

KCN scorpion toxins vary in length from 23 to 64 AAs with estimated molecular weights usually less than 4000 Da. As highly constrained polypeptides they adhere to either the inhibitor cysteine knot or disulfide-directed β–hairpin folding motif [35]. Extensive research was required in initial toxin studies to determine primary AA sequence, pharmacological target and establish three-dimensional structure. Proteolytic enzymes have been used to assist in defining critical disulfide bridging, with Edman degradation providing the location of these bonding pairs—a major undertaking with any multiple-disulfide bond-containing constituent. This was only possible once purified target materials were available, as achieved by paper and/or column chromatography [36].

Initially, X-Ray Crystallography was one of the few techniques available for establishing intricate details regarding molecular conformation. Results were slow, as crystallization techniques required specialized training, together with large amounts of pure sample [37,38]. Studies have shown that using racemic mixtures of L- and D-peptides has enhanced the ability to crystallize peptide toxins, improving the quality of molecular models [39]. Soon after, the power of NMR spectroscopy was realized beyond the standard small-molecule/organic analysis, and its application in peptide structure determination was embraced [40]. Using multi-dimensional NMR spectroscopy (500 MHz) it was determined that most scorpion peptide toxins adhere to a generalized α/β (scaffold) structural conformation that includes a characteristic number and location of α-helices and β-sheets [41,42,43,44]. Interestingly, using Maurotoxin (*Scorpio maurus palmatus*; MTX), Faljoun *et al*. showed that point mutations could be made which shifted the disulfide bridge framework, without altering the overall α/β scaffold of the toxin [45]. This revealed that the conserved α/β scaffold conformation was independent of toxin chain length, primary sequence and ion channel specificity, but importantly, that disulfide bridge patterns were paramount in tertiary structure stabilization and therefore pharmacological activity [45,46,47]. 

**Figure 2:**
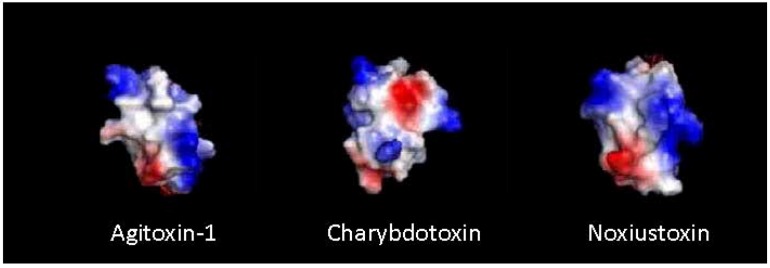
Computer models illustrating electrostatic distribution for three K^+^ channel (KCN) scorpion toxins. Adapted from Kumar *et al.* 2011 [49].

This was followed by the first computer based modeling, which was rudimentarily effective at predicting three dimensional structures by comparing homology sequences with previously defined toxin structures. Using graphics programs (*i.e.*, FRODO), the quality of analysis was a reflection on the power of the hardware/software of the time, and not necessarily the data [38]. Advancement in both computer systems and modeling software has significantly impacted the quality of current models [48], which can now incorporate electrostatic distribution and structural constraints (See Figure 2), characteristics of paramount importance in receptor docking [49]. The modeling of bi-molecular interactions between toxin and receptor proves invaluable in establishing peptide templates for advanced probe bioengineering [50,51,52].

First applied in the study of fundamental AAs and proteins in the late 1950’s [53] and early 60’s [54,55], mass spectrometric analysis of scorpion venoms remained undocumented until 1993 [56]. This technique revolutionized peptide research by providing instant mass analysis and sequence determination, while simultaneously minimizing sample requirements. Adding to this early work, advancements in mass spectrometry (*i.e*., Matrix Assisted Laser Desorption Ionization, MALDI-; Time of Flight, TOF-) have mirrored the exponential nature of toxin discovery by aiding in their isolation and identification. This is limited only by scaling parameters (signal to signal ratio) of the physical equipment being employed [57]. These approaches have advanced the structural determination of folding motif and the characteristic cysteine frameworks that help infer channel isoform selectivity [58]. Yet care must be taken in their analysis, as with these high-energy techniques, side chain functionality and PTMs are known to be susceptible to degradation under stringent conditions [59,60]. 

## 5. Synthetic Toxin Production

Major advancements in scorpion toxin development have been dependent on chemically synthesized toxins. Significant benefits of chemical assembly include the production of non-native analogs [61,62], truncated [63,64,65] or point mutated toxins [5,66], chimeric compounds [8,67], biotinylated molecules [5,68], toxins labeled with fluorescent moieties [69,70], pseudo peptides [71,72,73], or those containing D-amino acids [39,74], compounds which would prove difficult or impossible to produce using recombinant technology [75]. These approaches enhance functionality, utility and breadth of scorpion toxins in biomedical research.

The well-established synthetic approach pioneered by Merrifield, known as Solid Phase Peptide Synthesis (SPPS) [76], was first applied to a scorpion toxin with KCN activity in 1989. This resulted in the production of native-like material and truncated versions of NTX [65]. The biologically active, synthetic peptide was desirable for two reasons, (i) a large number of scorpions (~1000) would need to be milked in order to obtain quantities of material suitable for pharmacological experimentation (1 mg), as NTX represents only a fraction of the total protein (~1%) in the crude venom extract [26], and (ii) truncated versions of NTX do not occur naturally [65]. Noxiustoxin and the truncated terminal peptide segments were assayed and their pharmacological activity compared. Interestingly, NTX1-9 was toxic to mice through various routes of administration (intraperitoneal, subcutaneous or intraventricular), although necessitating application at higher doses (200 μg/10 g mouse weight) compared to native NTX (40 μg/ 20 g mouse weight) to achieve a parallel pharmacological response. 

Regardless of concentration, data showed that synthetic peptides retained biological activity and that their epitopes were capable of eliciting the desired pharmacological response, a concept currently being applied in cyclotide-epitope grafting for the production of novel molecular therapeutics [77] (Section 12). Simultaneously, both the solid-phase and solution-phase synthesis of ChTx were undertaken and completed [27,78]. SPPS inherently had direct advantages over solution-phase in terms of scheme complexity, use of less stringent reagents, and a single deprotection step [78]. SPPS exploits the ability to control the quality of peptide back-bone elongation by monitoring coupling yields using Ninhydrin following the addition of each successive AA residue [79]. This was not possible for the solution-phase synthesis as 5 segments were assembled individually and then ligated using water-soluble carbodiimide and one of two triazoles, either HOBt or HOOBt [78]. 

Additional advantages of SPPS become apparent when considering the incorporation of functional PTMs, which can be inserted into sequences thereby inferring native conformations, and resulting in the retention of biological activity (see below). There are however limitations to the synthetic production of peptides which become apparent when dealing with lengthy (>40 AAs), or extremely hydrophobic sequences, as seen in many scorpion toxins which act on Na^+^ channels. Long-chain synthetic scorpion toxins are difficult to fold due to the insolubility of the reduced form which leads to aggregation and the formation of polymeric material [75]. 

The initial KCN toxin studies focused on highly folded, tricyclic structures containing three disulfide bonds, with basic AAs arranged in clusters on the topical surface of the toxin [27]. The chemical synthesis of a new class of KCN toxins with four disulfide bridges was initiated when MTX was produced which displayed wide ranging activity on K_V_1.1, K_V_1.2 and K_V_1.3 isoforms [80]. Solid-phase strategies simplified sequential point mutation studies, otherwise known as Alanine scanning, which are commonly used to investigate key structural components of pharmacological activity (Section 7). In other cases, peptide toxin yields have been increased through the incorporation of chemically selective measures to direct disulfide bond formation using specially protected AAs (*i.e.*, Cysteine-ACM; acetamidomethyl) and the application of native chemical ligation (Section 11), potentiating the use of synthetic peptide toxins in a diverse range of research areas.

## 6. Recombinant Toxin Production

An alternate method for the production of peptide toxins is through the use of genetic engineering and recombinant technology. Typically this utilizes a modified bacterial expression system, with modified antibiotic resistance, used to promote the selection of replicating bacteria expressing the desired target material. Recombinant products obtained by genetic engineering have continued to improve our knowledge in the field of scorpion toxins and ion channels; however limitations exist mainly with low yields and at present, the lack of flexibility in the incorporation of non-native side chain functionality. 

Parallel to the initial KCN scorpion peptide assembly via SPPS, the first synthetic scorpion toxin gene was created, and transfected into common gram-negative bacteria, *Escherichia coli* [81]. The difficulties encountered in this early work included amino acid codon bias between mammalian and bacterial systems (organism specificity), and problems pertaining to the establishment of 3-Dimensional conformation, or correct disulfide bond connectivity [82]. Codon bias has been somewhat circumvented with the utilization of advanced *E. coli* cell lines (*i.e.*, BL21-CodonPlus(DE3)-RIPL; Agilent Technologies) infused with increased copies of specialized tRNAs [82,83], while challenges in disulfide bond formation have been overcome, to some degree, by the inclusion of protein disulfide isomerase to assist in folding [74,82,84]. 

The most significant hurdle experienced when using recombinant technology for the production of synthetic toxins is the inclusion of PTM’s, often critical components which influence folding, and establish toxin specificity and efficacy. Documented functionality of PTM’s in toxins is diverse, ranging from C*-*terminal amidation, carboxylation, phosphorylation and glycosylation to sulfonation, bromonation and hydroxylation [82,85,86]. Beyond C-terminal amidation, little is known about the extent and diversity of PTM’s in scorpion toxins with documented KCN activity. This area warrants additional investigation based on the potential to exploit side chain functionality in diversifying activity and application of prospective synthetic analogs for clinical use (see Section 14). 

This problem is being addressed presently through manipulation of genetic techniques to allow incorporation of non-coded AAs that display considerable chemical diversity [87,88,89,90,91]. Using synthetic tRNA’s, non-coded AAs have been incorporated with wide ranging functional prosthetic groups from redox-active AAs which modulate electron transfer [92,93], residues with ketone or azide functionality which aid in chemoselective bioconjugation [94,95], photocaged or photoisomerizable AAs which can photoregulate biological processes [96,97,98], metal binding AAs utilized in catalysis [89], fluorescent side chains which aid in localization and the study of channel dynamics [99,100,101], as well as native post translational modifications including hydroxylated and sulfonated residues [87,102]. Presently these approaches are absent in scorpion toxin production.

Assembly and purification of recombinant systems and scorpion toxins is lengthy and complex having a direct impact on rate of production, cost and yield. At present, SPPS represents a more flexible option in terms of creating complex toxins in large quantities, however, the advances made in the incorporation of non-native AAs, genetic engineering is developing momentum that will undoubtedly augment scorpion toxin research.

## 7. Structure Activity Relationship

Defining a toxin’s 3-Dimensional conformation, and further, the biologically active binding interface is an important part in understanding the structure activity relationship between toxin and receptor. This information becomes paramount when considering advanced peptide modifications and toxin bioengineering. Fundamentally, peptide toxins are short, single chain proteins interconnected by multiple disulfide bonds. The specific configuration of these disulfide bridges is indicative of compact folding and extraordinary molecular stability giving rise to target isoform selectivity and potency [42,47,71,103]. Several factors influence pharmacological activity in scorpion peptide toxins, these include: (i) primary sequence (encompassing charge and hydrophobic nature of residues); (ii) number and spacing of disulfide bridges; (iii) geometrical orientation of α-helices and β-sheets, and (iv) electrostatic and dipole orientation.

Initial structure studies examined single point mutants, created by scanning the primary sequence (excluding the cysteine framework) with the small, inert (in terms of R-group functionality), AA alanine, in an effort to isolate binding determinants while retaining native folding and structure characteristics. Each of the resulting alanine mutations can then be examined for pharmacological impact in terms of changes to *K*_d_, *K*_a_ or IC_50_. The first alanine scan through a KCN scorpion peptide was performed by Park and Miller (1992) on ChTx [104]. That investigation highlighted the importance of several surface reactive residues, most notable was Lys^27^, a highly conserved AA later identified as a critical pore-occluding characteristic in numerous KCN toxins [104,105,106,107]. These studies advanced the mapping of bimolecular interaction and demonstrated the powerful insights provided by the implementation of computer modeling [50,108]. At present there is a correlation (amongst the scorpion toxins) between dipole orientation and channel isoform specificity, while observed electrostatic features present in the contact surface influence toxin potency [49,109]. Figure 2 below demonstrates the capability of current computer modeling techniques and also illustrates electrostatic patterns which characterize KCN scorpion toxins.

Correlation between disulfide framework pattern, amino acid spacing and molecular target has been observed in scorpion peptides [61,62,71], as well as other classes of toxins from various natural sources (*i.e.*, Conus-[110]). KCN scoprion toxins do not deviatefrom this trend, with a wide range of disulfide framework patterns falling into five main classes as seen in Table 1. The manipulation of differential AA spacing and PTMs within these established KCN scorpion toxin familial disulfide frameworks should be undertaken based on the documented potential to harness this information for the bioengineering and production of synthetic templates for prospective molecular therapies of various human pathologies [7,42,71]. This approach has been broached in the production of a fully synthetic, successful peptide, Mokatoxin-1 (moka1), specific for K_V_1.3 and active in the regulation of immune T-cells (Section 14.2.3) [72]. As well as a disulfide variant of MTX (MTXPi1) whose disulfide bridge pattern has been altered without effecting the overall conformation of the peptide [46]. Interestingly, target affinity of the toxin was altered, despite the lack of change in the secondary structure.

**Table 1:** Established disulfide frameworks recognized within scorpion peptides with demonstrated KCN activity.

Class	Example	Disulfide connectivity	Target	Reference
I	KTX	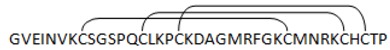	K_Ca_	[103]
II	CnERG1	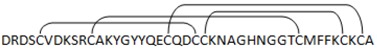	K_V_11	[111]
III	MTX	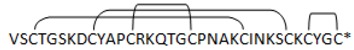	K_V_1, K_Ca_2, K_Ca_3.1	[46]
IV	HsTx1	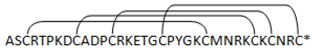	K_V_1.1, K_V_1.3	[45]
V	TtBut-Tx	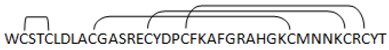	Shaker B	[112]

The above outlined disulfide frameoworks further represent a potential starting point for the design of novel, isoform specific probes which target directed channel isoforms. As potential probes, scorpion toxins have many advantages over primary antibody based detection systems, including specificity, and the ability to bind non-fixed tissue and cell samples. The inherent structure of KCN scorpion toxins makes them ideal candidates to be used as molecular probes for the multivaried investigation into ion channel function and physiology. Advanced bioengineering has further shown the potential clinical application of KCN scorpion peptides for the treatment of various human pathologies. 

## 8. Radiolabeled Scorpion Toxins and Receptor Localization

The development of anatomical channel localization studies and pharmacological displacement assays were facilitated by the development and production of radiolabeled derivatives of KCN scorpion toxins. Understanding both primary and secondary structure when designing and bioengineering KCN probes from scorpion toxin scaffolds is vital in order to maintain native conformation and in turn biological activity 

The first radiolabeled scorpion toxin developed was Iodinated ChTx, used to investigate the K_Ca_1.1 (MaxiK; BK) [113]. Native ChTx was isolated from crude venom and labeled with [^125^I]NaI using the IODO-GEN method [114] to produce [^125^I]ChTx, where monoiodination occurred at the C-terminal Tyrosine residue (Tyr^36^). This novel bioengineered peptide toxin probe demonstrated how chemical modifications can be made to the primary structure while maintaining topology of the secondary structure and retaining biological activity, thus expanding their applicability in research. Subsequently, the ability to radiolabel a synthetic peptide was demonstrated when Scyllatoxin, also known as Leiurotoxin-1 (*Leiurus quinquestriatus hebraeus*; ScyTx) was radiolabeled at His^31 ^(IODO-GEN method) and used for autoradiographic investigation of Small conductance Ca^2+^ activated K^+^ channel (SK) in rat brain sections [115].

The binding of the scorpion peptide Iberiotoxin (*Buthus tamulus*; IbTx) to K_Ca_1.1 depends on interaction of Tyr^36^ with the channel pore, resulting in the inability to utilize this commonly targeted AA for derivatization. Recombinant IbTx with a single point mutation, D19C, was iodinated using sulfhydryl reactive *N-*[^3^H]ethylmaleimide at the post-oxidative free thiol [116]. A two-point mutant (D19Y/Y36F) was also devised and produced recombinantly and the expressed peptide, IbTx-D19Y/Y36F, was iodinated at position Tyr^19^ ([^125^I]Na in the presence of Enzymobeads; Bio-Rad) [117]. The latter probe was successfully used to investigate the expression of K_Ca_1.1 in bovine tracheal smooth muscle sarcolemmal membranes.

As technology progressed, so too did the production of other radiolabeled KCN toxin-probes. They have been used to investigate the expression and distribution of a number of KCN isoforms in a wide range of cell types, contributing uniquely to understanding the role of KCN in membrane bioenergetics and their subsequent effects on human physiology. Table 2 illustrates the usefulness, diversity and progression of radiolabeled KCN probes, and in some cases the extent of bioengineering undertaken to achieve their potential as effective molecular calipers.

**Table 2:** List of radiolabeled KCN probes used in the investigation of receptor tissue localization/expression as well as electrophysiology in terms of binding kinetics.

Toxin	Modification	Name	Label	Target	Reference
ChTx	native	[^125^I]ChTX	[^125^I]-Y36	K_Ca_, K_V_	[113]
	R19C	[^3^H]-ChTx-R19C	[^3^H]-C19	K_Ca_, K_V_	[118]
ScyTx	F2Y	^125^I-[Tyr2]ScyTx	[^125^I]-Y2	K_Ca_	[119]
MgTX	native	[^125^I]MgTX	[^125^I]-Y37	K_V_1.2, 1.3	[120]
IbTx	V5Y, Y36F	[mono-iodo-Tyr5, Phe36]-IbTx	[^125^I]-Y5	K_Ca_1.1(BK)	[121]
	D19C	[^3^H]-IbTx-D19C	[^3^H]-C19	K_Ca_1.1(BK)	[116]
	D19Y/Y36F	[^125^I]IbTx-[D19Y/Y36F]	[^125^I]-Y19	K_Ca_1.1(BK)	[117]
BeKm-1	native	[^127^I]-BeKm-1	[^127^I]-Y11	K_V_11.1(hERG)	[122]
HgTx	A19Y/Y37F	^125^I-HgTx_1_-A19Y/Y37F	[^125^I]-Y19	K_V_1.1/2/3/6	[123]
BmTX3	native	^125^I-sBmTX3	[^125^I]-Y37	A-type current	[124]
NTX	native	[^125^I]Noxiustoxin	[^125^I]-Y37	K_V_1.1	[125]
KTX	KTX(1-37)	^125^I-KTX(1-37)	[^125^I]-H34	K_Ca_	[126]
Lqh III	native	[125I]Leiurutoxin III	[^125^I]-Y8	K_V_1.1	[125]

## 9. Bioconjugation

In recent years there has been a paradigm shift moving away from the use of radiolabeled toxins due to the inherent difficulties and dangers associated with employing radioactivity. However the need persists for the study, and incorporation of ion channel physiology into human pathological disease models. In turn, this has led to major advancements in the design and chemical synthesis of fluorescently bioengineered toxin-probes that have alleviated many of the issues experienced when working with radiolabeled toxins. 

Stimulated by the introduction of novel chemical strategies for the attachment of varying prosthetic groups, a process referred to as bioconjugation, as well as a growing selection of available fluorophores, covalent and ionic (biotin-streptavidin complexes) techniques have been developed to bioconjugate fluorescent moieties to KCN scorpion toxins for the classical purpose of detecting and localizing ion channel isoforms *in vivo*, as illustrated by ChTx-biotin [68]. Initially, basic strategies developed with standard *N*-terminal derivatization, and advanced to modification of the ε-amino functionality of introduced lysine residues (amine chemistry) [127,128,129]. Alternatively, yet with its own unique set of conditions and difficulties, the use of thiols as targeted conjugation sites have gained popularity (thiol chemistry) [69,118,130]. Another approach currently being developed for fluorescent incorporation is phosphate-azide “click” chemistry [131,132,133].

In 1993, Robitaille *et al*. employed modified bioconjugation strategies previously applied to the conotoxin ω-CgTx [134,135] to produce the first biotinylated scorpion toxin with directed KCN activity [68]. Prepared via SPPS, ChTx-biotin was created via direct chemical conjugation of Succinimidyl-6-(biotinamido)hexanoate to the exposed *N*-terminal [68]. The ChTx-biotin probe was used to investigate the distribution of Ca^2+^-gated K^+^ channels in relation to Ca^2+^ channels and release sites of transmitters at the neuromuscular junction. Shimony *et al*. (1994), used a bacterial expression system to produce a folded biologically active mutant of ChTx (*i.e.*, ChTx[R19C]). This novel bioengineered peptide retained an unpaired “spinster cysteine” for derivatization based on thiol directed chemistry [118]. The point of the insertion was determined by in-depth structural analysis, and ChTx[R19C] was produced with the strong nucleophilic thiolate mutation made at a location far from the interactive surface, again in order to retain functional activity of the toxin during the bioconjugation of fluorescent labels. 

Attributed partly to the entrenched use of primary antibodies in the scientific culture, combined with potential difficulties in constructing and purifying fluorescent bioengineered peptide probes, most have been used recently in the periphery in ion channel research. Recently however, limitations in antibody technology, in terms of dealing with live-cell processes, are appearing which represent obstacles in advancing live-cell visualization. There is a growing desire for advanced imaging and localization technology, this requirement has emerged specifically from the increased need to investigate real-time physiological processes *in vivo*, including channel protein translation, trafficking and surface expression. 

In 2002, a fluorescent version of Hongotoxin (*Centruroides limbatus*; HgTx) was produced synthetically [69], and subsequently used in receptor localization experiments utilizing ultrasensitive microscopy known as the single-dye tracing technique [136]. A number of different fluorescent moieties were successfully conjugated to the bioactive toxin while simultaneously maintaining potency, namely Cy3, Alexa488 and Alexa546, although a number of other dyes were also conjugated with varying degrees of success. From this study, it was determined that both positioning within the toxin as well as selection of fluorophore are crucial to producing successful fluorescent-toxin probes. The resulting fluorescent toxin was proficient in localizing K_V_1.3 in Rat brain sections [69], as well as Jurkat cells at the single molecule level [136]. 

**Figure 3:**
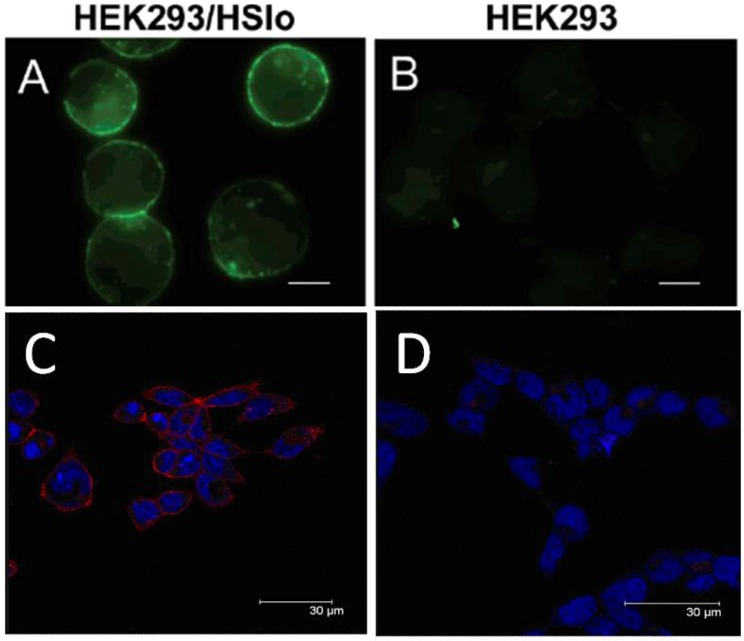
(Top) Original epifluorescent images adapted from Bingham *et al*. 2006 [5]. (**A**) Stably transfected HEK293/hslo; (**B**) Wild type HEK293. (Bottom) Advanced confocal imaging of experimentally identical cell lines (HEK293/hslo, and HEK293) with the fluorescent peptide IbTx[D19K]-LC-biotin [137]. Experiments were heavily controlled (as specified in Bingham *et al*. 2006 [5]) for non-specific binding and are available by request; (**C**) Stably transfected HEK293/hslo (visualized with streptavidin Alexa Fluor conjugate-red); (**D**) Wild type HEK293.

Refuting common conception that amine modification of lysine destroys pharmacological activity of scorpion toxins targeting K^+^ channels [68], Bingham *et al*. (2006) synthesized a biotin derivative of IbTx by replacing aspartic acid (Asp^19^), with the non-native AA *N*-ε-(d-biotin-6-amidocaproate)-L-lysine (Anaspec; IbTxD19K-LC-Biotin), incorporating a spacing component, or “linker” which provided steric clearance from the peptide backbone. This eliminated perturbation of the three-dimensional tertiary structure of the toxin, which was essentially locked-in by the disulfide framework. Coupled with the secondary fluorophore, Alexa488-Streptavidin (Molecular Probes), the probe enabled visualization of BK (*hslo*) channel in stably transfected HEK293 using standard epifluorescent microscopy [5]. Akcan *et al*. (2012) further substantiated this bioengineering approach with the bioconjugation of a near infrared fluorophore (NIRF) to lysine 27 of Chlorotoxin (*Leiurus quinquestriatus quinquestriatus;* CTX) which dramatically increased serum half-life when assayed [129]. From this groundwork, *in vivo* channel localization and visualization can be advanced exponentially with the application of superior imaging techniques (*i.e.*, confocal microscopy; see Figure 3). Use of such bioengineered toxins allows for the visualization of cellular surface details, including banding and patch like clustering of ion channels in expressing cells [5,68].

To demonstrate the utility of fluorescent KCN probes the most compatible toxins and bioconjugation chemistry have been utilized in the process of manipulation and structural bioengineering of peptide toxin candidates. This work has allowed for the investigation of up- and down-regulation of receptor subtypes in connection with various human pathologies and disease states. A complete list of fluorescent KCN toxins can be seen in Table 3.

**Table 3:** Table of fluorescently labeled peptide scorpion toxins specific for various potassium channel isoforms.

Toxin base sequence	Mutation	Bioconjugate	Target	Reference
ChTx	*N*-term	ChTx-biotin	K_Ca_, K_V_	[68]
	R19C	Rhodamine-ChTx-R19C	K_Ca_, K_V_	[118]
HgTx	A19C	HgTX_1_-A19C-Cy5	K_V_1.1/2/3/6	[69]
		HgTX_1_-A19C-Cy3	K_V_1.1/2/3/6	[69]
		HgTX_1_-A19C-Alex488	K_V_1.1/2/3/6	[69]
		HgTX_1_-A19C-Alex546	K_V_1.1/2/3/6	[69]
		HgTX_1_-A19C-Alex594	K_V_1.1/2/3/6	[69]
		HgTX_1_-A19C-Alex660	K_V_1.1/2/3/6	[69]
		HgTX_1_-A19C-Alex680	K_V_1.1/2/3/6	[69]
		HgTX(1)-A19C-Cy5	K_V_1.1/2/3/6	[136]
IbTx	D19C	IbTx-D19C-Alexa488	K_Ca_1.1(BK)	[70]
	D19K	IbTx-LC-biotin	K_Ca_1.1(BK)	[5]
		IbTx-LC-FAM	K_Ca_1.1(BK)	[138]

## 10. Scorpion Toxin Chimeras

A novel approach for determining the structural components that define scorpion toxin isoform selectivity in KCN is the design, bioengineering and investigation of chimeric peptide toxins. This approach was initiated when IbTx and ChTx were targeted as chimeric candidates due to their differential selectivity for KCN isoforms, K_Ca_1.1 and K_V_1.3 respectively, and high AA homology (~68%), in the presence of a conserved disulfide framework. The major consideration in choice of peptide candidates was the demonstrated selectivity of IbTx (K_Ca_1.1), and the promiscuity of ChTx, which inhibits both K_Ca_1.1 and K_V_1.3. Thus combining individual block sections of AA sequences/secondary structural features was a natural development for defining their unique pharmacological properties. The resulting chimeric toxin revealed that both *N*- and *C*-terminal domains are central in governing isoform selectivity, demonstrating that terminal sequences can be modified to induce artificial interactions [8].

A metal binding chimera of ChTx was designed and synthesized to examine the extent of modification possible in scorpion toxin scaffolds [139]. Nine residues, based on the Zn^2+^ binding site of carbonic anhydrase B, were inserted into the native ChTx sequence. The resulting chimera successfully binds metal ions confirming the functional versatility of the scorpion toxin scaffold. This provides a template for the assembly of conformationally constrained peptide libraries with both therapeutic and industrial potential.

Acetylcholine receptors (AChR) have been identified as potential clinical targets as their binding evokes flaccid paralysis, and important characteristic in anesthetics [140]. Certain snake venoms contain curaremimetic neurotoxins which bind to the AChR at the postsynaptic membranes of skeletal muscle including Toxin a, from the venom of black-necked spitting cobra (*Naja nigricollis*). The small disulfide-stabilized structure of the scorpion toxin ChTx was used as a basic scaffold for the active residues of loop II in Toxin a, corresponding to the central part of the curaremimetic site [141]. The chimera represents a less toxic, yet equally effective immunogen for the production of toxin neutralizing antibodies, an important element in vaccine design and production. The rationale design of synthetic vaccines could be improved significantly by utilizing this approach, resulting in increased accessibility through decreased costs.

Several Noxiustoxin-Iberiotoxin (NTX-IbTx) chimeras were developed to investigate how the length of the α-carbon backbone, and differences in the α/β turn configuration contribute to target isoform selectivity [62,67]. Results indicate that backbone elongation alters steric interaction with the channel pore, while differences in the α/β turn alter the geometry of the toxin-binding surface. By tailoring toxin size, and geometric orientation of charge distribution, peptides can be designed to discriminate between KCN isoforms based on vestibule architecture. 

Another peptide toxin chimera, Tsk-MTX, has been structurally bioengineered and identified as a lead prototype scaffold for synthetic toxin-probes that could be designed *de novo* to produce potent blockers with unique specificity and/or affinity toward targeted KCN isoforms [142]. The 38 residue peptide scaffold was derived from MTX, a 34 AA residue peptide cross linked by four disulfide bonds, active on both K_V_ and K_Ca _isoforms [80], and TsKapa (*Buthidae Tityus serrulatus*; *Tsk*), a 35 AA residue peptide cross linked by 3 disulfide bonds and active only of K_Ca_ channels [143]. The critical difference impacting target specificity between the two toxins is the presence of either two or three β-sheets respectively.

In its native form, CTX is active on small conductance Cl^−^ channels and displays no affinity for K^+^ channels. It has been widely used as a Cl^−^ channel blocker as well as a glioma specific marker with diagnostic and therapeutic potential [144]. CTX has structural characteristics in common with α-KTx peptide toxins including Lys^27^ and a similar α/β scaffold. A CTX-AgTx2 chimera was constructed from the base scaffold of CTX, and three residues composing the β-sheet of Agitoxin-2 (*Leiurus quinquestriatus*; AgTx2) which infers K_V_1 specificity [145]. This work showed that structural modifications could be made successfully which induce non-native inhibitory properties, an exciting prospect for protein design and engineering. 

In an effort to determine whether increasing the molecular contacts between a toxin and an ion channel impacts affinity, M’barek *et al*. (2005) used a gain of function approach to evaluate this hypothesis [46]. A chimeric peptide toxin was developed based on the template of MTX_Pi1_, a disulfide variant of Maurotoxin. The *N*-terminal of “native” MTX_Pi1 _was replaced with that of Butantoxin (*Tityus serrulatus*, *Tityus bahiensis*, *and Tityus stigmurus*; BuTX), a 40 AA peptide constrained by 4 disulfide bridges and specificity for K_V_1.2 with an IC_50_ = 165 nM [46,146]. Transfer of 9 *N*-terminal residues from BuTX to MTX_Pi1_ resulted in a novel chimeric peptide with 5 disulfide bridges. The active toxin BuTX-MTX_Pi1 _displayed a 22-fold greater affinity for K_V_1.2 than “native” MTX_Pi1_, revealing that increasing the molecular contacts between toxin and receptor, can significantly enhance affinity for KCN target isoforms, an interesting concept in terms of bioengineering.

The chimeric peptide, AgTx2-MTX, was produced synthetically consisting of a truncated Agitoxin-2 sequence which targets K_V_, conjugated with the complete Maurotoxin sequence which targets both K_V_ and K_Ca _type channels [7]. Interestingly, the chimeric peptide displayed uncharacteristic disulfide bridging patterns, producing a more potent peptide than either parent toxin, and found to be active of K_V_1.2. This is yet another example of the applied use of synthetic-bioengineered peptide toxins as viable molecular probes for studying varying KCN isoforms characteristics. 

## 11. Native Chemical Ligation

Native chemical ligation is a powerful synthetic strategy allowing peptide synthesis to move beyond the constraints of the standard length linear SPPS (~50–60 AAs) [147]. Proven by the chemical and semi-synthesis of large functional proteins (>10 kDa), including the ion channel KcsA [148,149], this strategy has also facilitated the incorporation of small synthetic peptide fragments into larger recombinant proteins, allowing the integration of various non-native AAs and fluorophore derivatives [39,150]. 

In the past, SPPS methodology has been partially restricted by the difficulty of ensuring complete N^α^-deprotection and AA acylation throughout a long peptide chain, especially in syntheses over 50 residues [151]. This limitation is mostly attributed to their length, hydrophobic nature and high degree of cysteine content. Alleviating these issues, native chemical ligation employs a C-terminal thioesterified peptide that is chemically ligated to a peptide bearing a free *N*-terminal cysteine, this occurring sequentially using small manageable size linear peptide blocks. This process was initially performed principally using Boc-SPPS [151], however Li and co-workers described a novel approach leading to the production of a peptide thioester via Fmoc-SPPS [152]. Unfortunately difficulties remained, as this method produced significant aminolysis of the primary peptide thioester species. Methodologies have been sequentially improved, demonstrated by the synthesis of the scorpion toxin II Hexapeptide [151]. 

In 2009, using strategies developed for native chemical ligation, Bingham *et al*. produced a bioengineered, two point mutation of Iberiotoxin with greatly increased yields (as much as 12-fold over standard linear synthesis). This increase can be attributed to the ease of synthesis and purification, a consequence of limiting side-reactions which functionally reduces accumulation of stepwise deletions, hydrophobic aggregation, and disulfide cross-linking [153]. All of which are common occurrences encountered during extended syntheses which detrimentally affect yield. Native chemical ligation also proves useful when seeking to generate racemic mixtures of peptide toxins to ease the production of crystals for X-ray determination of overall three-dimensional toxin structure. Synthetic D-kaliotoxin and L-kaliotoxin enantiomers (*Androctonus mauretanicus mauretanicus*; KTX) were synthesized by NCL and used to produce highly ordered racemic crystals, which facilitated X-ray structural determination [39,64]. Crystallization proved difficult when solely employing the L-protein isoform.

This synthetic approach provides a straightforward method for the preparation of multiple milligram quantities of peptide toxins, required for the production of superior quality electron density maps and structural models with lower error. This information is extremely valuable in the design and bioengineering of future KCN-specific scaffolds. Furthermore, this approach is successfully being utilized in the production of cyclized toxins for the development of potential molecular therapeutics [154,155], see Section 12. 

## 12. Peptide Backbone Cyclization

Scorpion toxin scaffolds specific for K^+^ channels have tremendous potential in rational drug design and development [156], see Section 14. Unfortunately, they suffer from a number of disadvantages *in vivo* owing to their inherent peptidic structure including proteolytic and thermal degradation, as well as poor bioavailability. Peptide backbone cyclization, or *N*- to *C*-terminal ligation, is capable of dramatically increasing this therapeutic potential by minimizing aforementioned drawbacks [157,158]. To achieve this, several factors must be considered in regard to the toxin candidate including, (i) distance between termini; (ii) relative orientation of termini; (iii) flexibility of the termini; (iv) position of disulfide bonds and (v) understanding of key residues involved in biological activity or binding [159]. This bioengineering approach has thus far proven successful exemplified by the production of *in vitro* fluorescent probes [129], the design of therapeutic scaffolds for epitope chimerization [160], and the re-engineering of conotoxins for the treatment of neuropathic pain [161].

Naturally occurring in plants, animals and bacteria, circular peptides known as cyclotides are hypothesized as a natural component in host defense systems. Displaying a diverse range of biopharmaceutical properties including anti-HIV, antimicrobial and insecticidal activities [77], cyclotides are inherently stable, surmounting many of the primary obstacles observed when bioengineering peptides as potential drug therapies [159]. Structurally, cyclotides are comprised of a circular peptide backbone, and a disulfide framework commonly referred to as a cysteine knot motif [162,163]. Using techniques developed for native chemical ligation [164], several therapeutic, acyclic toxins have been cyclized, including the scorpion peptide CTX [158], and the χ-conotoxin MrIA, the latter of which has subsequently entered clinical trials as a potential treatment for neuropathic pain [165,166].

**Figure 4:**
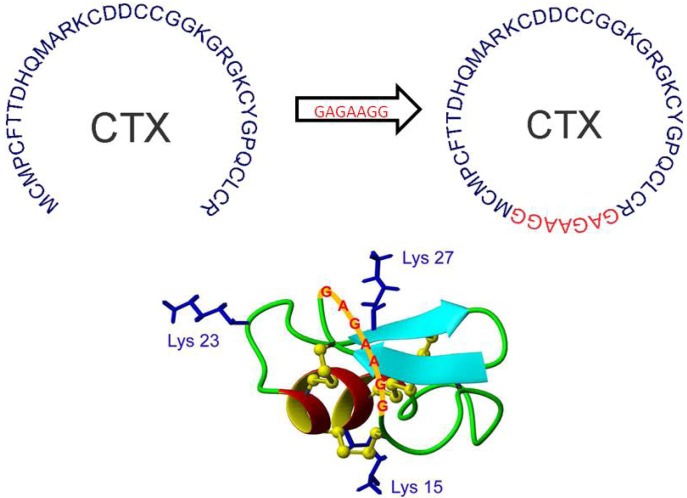
Cyclization of the scorpion peptide Chlorotoxin (CTX). A flexible 7 residue linker (GAGAAGG) was used to bridge the ~11.7 Å gap between the *N*- and *C*-terminals. Adapted from Akcan *et al*. 2011 [129].

As with other peptide toxins, cyclotides can be bioconjugated to fluorescent moieties, however in this form *N*-terminal labeling is not possible and limitations are presented by the sterics of the parent sequence backbone. Potentially, topologically accessible lysine residues can be used, or can be inserted into functionally inert region of the parent sequence, as discussed in Section 9. Akcan *et al*. (2011) attempted to determine if fluorophore bioconjugation and cyclization of Chlorotoxin (CTX) would improve the stability of CTX bioconjugates by eliminating the accessibility of terminal AAs to circulating peptidases. Utilizing native chemical ligation, Cyclic-CTX was synthesized by Boc chemistry. A seven-residue linker (GAGAAGG) was required for cyclization (see Figure 4) due to the proximity of the termini (11.7 ± 1.5 Å) [129]. Glycine and alanine were chosen for the linker due to their small size, and based on the premise that side chain functionality would not perturb the native three-dimensional conformation of the parent peptide backbone. Later bioconjugation of near infrared fluorophores to create fluorescent probes increased serum stability of cyclic toxins from 70% to 90% (after a 24h incubation) [129]. 

The ability to deter proteolysis represents a major bioengineering advancement when viewing peptides as molecular therapeutics. However, consideration must be given to efficacy, pharmacokinetics, pharmacodynamics and toxicity—which are influenced by a peptide’s ability to retain its structural integrity. As reported earlier, serum stability of CTX was subsequently improved by peptide bioengineering. This suggests that proteolytic cleavage can be reduced following fluorescent bioconjugation and cyclization, while *in vivo* excretion and metabolism remain unaltered [129]. This positive attribute infers that conformational stability will facilitate oral administration, while not changing pharmacokinetic and pharmacodynamic (PKPD) parameters. This advances bioengineering peptide therapeutics, making them biologically safe, when compared to their non-cyclic counterparts—this likely having an impact on how we view the future progression of scorpion toxin therapeutics. 

Augmenting this technology, using cyclic peptide backbone frameworks in a process known as grafting, synthetic therapeutics can be bioengineered or ‘stitched together’ with pre-determined targets, much like chimeric toxins (Section 10). This has developed into what has become one of the most successful forms of rational drug design to date. This approach combines small bioactive peptide epitopes with known molecular scaffolds, stabilized by cyclic frameworks thereby increasing oral bioavailability and potency [155]. This approach has been verified by grafting analogs of the plant peptide Kalata B1 [160]. Cyclization and grafting techniques now present the opportunity to re-engineer medically relevant KCN scorpion toxins (Section 14) to produce stable, directed, molecular therapeutics with novel KCN targets.

## 13. Potassium Channels as Clinical Targets

Potassium channels have been implicated in a number of human pathologies such as Asthma [2], Cardiac Arrhythmia [167], T-cell mediated autoimmune disease [73,167], immune response to infection and inflammation [168], and Hypertension [169]. The bioengineering of scorpion peptides represents a valuable area of research, and a potential pool of bioactive scaffolds for the development of novel drugs used in the treatment of these various disease states.

### 13.1. Asthma

According to the Center for Disease Control and Prevention over 18 million people (~8% of the U.S. population) are affected by asthma. It is one of the most common long-term diseases in children, affecting ~9% of all children (roughly 7 million). Usually treated with corticoid steroids, this approach is becoming ineffective in a growing number of those afflicted.

Two potassium channels (K_V_1.3 & K_Ca_3.1), expressed in non-excitable cell types (K_V_1.3: T-cells; K_Ca_3.1: mast cells, macrophages, airway smooth muscle cells, fibroblasts and epithelial cells), have emerged as potential targets as these KCN isoforms are widely distributed in immune and structural airway cells, playing a key role in cellular activation, proliferation and migration, major factors in asthma pathophysiology [2]. These anatomical characteristics make small molecule blockers an obvious choice for potential therapeutics. Margatoxin (*Centruroides margaritatus*; MgTX) inhibits K_V_1.3 with a *K*_d _= 110 pM [120], while ChTx and MTX inhibit K_Ca_3.1 with respective *K*_d_’s of 5 nM and 1 nM [2]. Non-specific binding of these toxins to K_Ca_1.1 and K_V_1.3 (ChTx) and K_V_1.2 (MTX) pose a problem, as does administration. 

Chimeric modeling (Section 10) may resolve issues relating to specificity, while cyclization (Section 12) could potentially improve oral bioavailability. This hypothesis developed based on the orally active K_V_1.3 blocker ICA-17043 (Senicapoc), (Icagen Inc., Durham, NC, USA), which inhibits late airway response and the development of bronchial hyperresponsiveness in sheep-asthma models [170].

### 13.2. Cardiac Arrhythmia

A host of KCN isoforms including K_V_1.5, K_V_4.2, K_V_4.3, K_V_7.1, and K_V_11.1 have been implicated for their involvement in a number of cardiac arrhythmias such as atrial fibrillation [171], Long QT syndrome [172] and torsade de pointes [167,173]. Generically defined as any irregularity in the normal activation sequence of the myocardium, arrhythmias and resulting sudden cardiac death are responsible for 600,000 deaths each year in the United States alone [174]. Unfortunately, many currently available prescription drugs also elicit side effects that result in varying arrhythmic physiological states. In light of this, the United Stated Food and Drug Administration requires pharmaceutical companies to perform cardiovascular toxicity testing on all investigational new drugs [175]. 

A recent survey of 29 pharmaceutical companies showed that 93% based their assessment of ventricular repolarization risk solely on a K_V_11.1 competitive binding assay [176]. One highly selective scorpion toxin, although known to bind the outer vestibule of K_V_11.1, could potentially be re-engineered as a valuable probe for the investigation of hERG associated cardiac arrhythmia [177] and a potential screen for investigational new drugs.

### 13.3. T-Cell Mediated Autoimmune Diseases

T-cell mediated autoimmune diseases include Multiple Sclerosis, Type-1 diabetes, rheumatoid arthritis, and psoriasis [167]. Investigators in the Merck Laboratories used the scorpion peptide, MgTX to show that K_V_1.3 blockade can inhibit immune response *in vivo *by depolarizing the T-cell membrane, effectively reducing the driving force for the entry of Ca^2+^ through the calcium-release activated Ca^2+^ channel. K_V_1.3 channel blockers also selectively inhibit Ca^2+^ signaling, proliferation, and *in vivo* migration of CCR7^−^ effector memory T-cells and therefore act as immunomodulators as opposed to immunosuppressants (which have their own obvious drawbacks). Interestingly, blockade of K_V_1.3 by MgTX is the impetus for the translocation of the glucose transporter, GLUT4, to the plasma membrane which functionally improves sensitivity to insulin [178]. Some inherent problems with bioengineering scorpion toxins for the treatment of K_V_1.3 channelopathies include (i) the short half-life of scorpion toxins—which could potentially be overcome by cyclization (Section 12), or addition of stabilizing side chain functionality; (ii) the differential expression of KCN isoforms (human vs. mouse)—which could be resolved using chimeric toxins in the well established mouse model of autoimmune disease; and (iii) toxin-channel specificity [167].

The α-KTx family of scorpion toxins represents an attractive collection of compounds, which target K_V_1.3. This family can be broken down into two distinct groups, A and B, where group B toxins have a truncated *N-*termini and two site specific residues (His^9^ and Asp^33^) which account for the functional divergence between these groups [73]. These scorpion toxins could potentially serve as structural templates used to bioengineer novel molecular therapies for the treatment of K_V_1.3 channelopathies such as T-cell mediated autoimmune disease. 

### 13.4. Immune Response to Infection and Inflammation

The voltage-gated ion channels K_V_1.3 and K_V_1.5, which constitute the main KCN assemblage in macrophages, are involved in the activation and proliferation of leukocytes. Proliferation and activation of which trigger an induction of the outward K^+^ current that is under transcriptional, translational, and posttranslational control [168]. Macrophages are phagocytes that serve also as antigen-presenting cells producing inflammatory and immunoactive substances which modulate the immune response: A process that is functionally stimulated by hormones and cytokines. KCN blockade by specific antagonists decreases macrophage production and proliferation thereby reducing the activation of cytokines and reducing the presentation of antigens to T-lymphocytes [179].

The major KCN isoform expressed in macrophages is a tetrameric K_V_1.3/K_V_1.5 hybrid. Currently, no hybrid K_V_1.3/K_V_1.5 toxin is known to exist, therefore molecular therapies would require the design of chimeric toxin based on K_V_1.3/1.5 pore characteristics. The toxin Vm24 (*Vaejovis mexicanus smithi*), could potentially be utilized as a base scaffold due to its extreme affinity for K_V_1.3 (*K*_d_ = 2.9 pM) [180].

### 13.5. Hypertension

The Ca^2+^-activated K^+^ channel, K_Ca_1.1, from the gene *slo*, functionally regulates smooth muscle tone in pulmonary airways and vascular beds [5,169]. This is accomplished by facilitating feedback regulation against the rise of intracellular Ca^2+^, membrane depolarization and vasoconstriction that in turn promotes outward K^+^ current, initiating membrane hyperpolarization [181]. Due to this regulatory role in vascular tone, K_Ca_1.1 has been identified as a molecular target for the treatment of hypertension.

Scorpion toxins have been classified which act on K_Ca_1.1, as first demonstrated by ChTx [6]. Unfortunately ChTx displays promiscuous behavior cross reacting with other KCN isoforms, making it less than ideal as a therapeutic candidate. Interestingly, two short chain scorpion toxins, IbTx [5], and BmTx3B [182], in addition to one long chain scorpion toxin BmP09 [183] are well documented, selective inhibitors of K_Ca_1.1. While most long chain scorpion toxins are known to be specific for Na^+^ channels, the sulfoxide produce by Met^66^ in BmP09 induces a drastic shift in target specificity [183]. Chimeric bioengineering could elucidate structural characteristics responsible for this shift in specificity towards K_Ca_1.1, creating a highly specific, and potent toxin scaffold that could hypothetically be cyclized for therapeutic application. 

Other human pathologies have been associated with varying KCN isoforms including links between K_V_1.1/2 and episodic ataxia, partial seizures and myokymia disorders [184], K_V_3.4 and Alzheimer’s [185], K_V_7.2–7.5 and epilepsy [186], as well as K_V_7.2–7.5 and the treatment of neuropathic pain [187], but are beyond the scope of this review. The link between these diseases and KCNs remains an important area of research and treatments are being investigated. Using some of the techniques highlighted here, various scorpion toxins (BTK-2 and OdK1, Episodic Ataxia [49,188]; sBmTX3, Alzheimer’s [189]) could prove invaluable as molecular scaffolds for the development and bioengineering of molecular therapeutics for the treatment of these debilitating human pathologies.

As evidenced, a number K^+^ channels have been identified as a possible therapeutic targets for various human pathologies. Using advanced techniques in peptide bioengineering, as outlined above, scorpion toxins could potentially play a major role of the development of future biopharmaceuticals.

## 14. Potassium Channel Toxins in the Clinic—Bioengineering Molecular Therapeutics

### 14.1. HIV/AIDS

#### 14.1.1. Charybdotoxin

In 2007, the Center for Disease Control estimated almost 36,000 new AIDS cases in the United States alone. This number contributes to the nearly half-million people already living and dealing with this stigmatic autoimmune disease. These numbers fail to account for the 1.2 million HIV infections, a retroviral infection which has yet to develop into full-blown AIDS. HIV/AIDS cases in the United States pale in comparison to the staggering numbers documented in Sub-Saharan Africa, most notably, South Africa [190]. Current treatments, although successful, report significant adverse side effects including nausea, fatigue, emotional distress and headaches [191]. The development of novel therapies could enhance the quality of life for those individuals receiving treatment for HIV/AIDS infections.

A ChTx based miniprotein was used in phage epitope randomization studies to identify synthetic peptide constructs that bind gp120 and block the gp120-CD4 interaction. Inhibiting this mechanism stops infection by binding CD4 with gp120 and preventing CD4-induced conformational isomerization which initiates co-receptor binding and viral cell fusion [192]. This technology may have potential therapeutic value for the treatment of HIV infection and AIDS.

TXM1, is a novel bioengineered peptide mimetic based on ChTx, where the CD4 CDR2 loop sequence, Gln-Gly-Ser-Phe (40QGSF43), was substituted for residues 24–27 in the β-turn of ChTx [192]. Experimentally, the ChTx chimera/FLAG gene was assembled by T7 DNA polymerase extension and found to bind selectively to gp120 (ELISA competition and biosensor direct binding assays).

These results demonstrate that CD4 recognition mimetics can be bioengineered within a β-turn of the ChTx miniprotein scaffold, a development which illustrates that scorpion toxins can be synthesized to contain epitopes imitating the cell receptor CD4. With a diverse pool of scorpion toxin scaffolds available with similar α/β motifs, the extension of the phage randomization approach to other scaffolds in this family could fuel the development of a number mimetic antagonists suitable for the treatment of HIV/AIDS as well as other debilitating human pathologies.

#### 14.1.2. Scyllatoxin

In another example of chimeric scorpion toxin-epitope assembly for the treatment of HIV/AIDS, nine residues from CD4, vital to the binding of HIV-1, were inserted into a homologous region (β-hairpin loop) of ScyTx in a three step process [193]. This was functionally achieved by transferring the side chains of the CD4-gp120 binding interface to a structurally equivalent region of the ScyTx scaffold.

The small size of these miniproteins infers many advantages including ease of synthesis and manipulation, including the ability to incorporate fluorophores and non-native AAs. This technology is especially valuable due to the large size of the intricate CD4 binding surface which usually makes the rational bioengineering of small mimetics, representing these interfaces, a daunting task.

### 14.2. T-Cell Mediated Autoimmune Disease

#### 14.2.1. OSK-1[E16K, K20D]

Bioengineered OSK-1[E16K, K20D] (IC_50_ = 3 pM) is a synthetic toxin based on the template sequence of OSK-1 (*Orthochirus scrobiculosus*; *α*-KTx3.7) displaying an increased affinity (~5×) for K_V_1.3 over the native peptide (IC_50_ = 14 pM) [194]. Several residues (Arg^12^, Glu^16^, Lys^20^ and Thr^36^) were explored for their impact on toxin-receptor pharmacology. Two mutations were ultimately introduced into the native sequence during Fmoc-SPPS, E16K and K20D, having the greatest impact on affinity. The synthetic bioengineered OSK-1[E16K, K20D] toxin retains biological activity towards other channel isoforms (K_Ca_3.1), a major drawback for a potential clinical therapeutic.

#### 14.2.2. ADWX-1

Several T-cell mediated autoimmune diseases are mediated by K_V_1.3 including multiple sclerosis, Type-1 diabetes, rheumatoid arthritis, and psoriasis [195]. A potent, and highly selective inhibitor of K_V_1.3, the molecular therapeutic ADWX-1 (IC_50_ = 1.89 pM), was designed based on the structure of BmKTX (*Buthus Martensi*) [195,196]. Three non-native mutations were engineered into ADWX-1 (G11R, I28T, and D33H) which increases its affinity 100-fold over native BmKTX (*K*_d_ = 0.2 nM). Several factors were considered in this modern, and poignant example of rational drug design including distribution of peptide functional residues, residue polarity, and especially the negatively charged residues known to be intimately involved in peptide docking [73].

ADWX-1 was rationally designed in a three-step process, aided by pharmacological data obtained by alanine-scanning mutagenesis and computational modeling (Section 7). Step1: The distribution of negatively charged residues in the BmKTX peptide template were adjusted in order to increase proximity of Lys^26^ to the channel mouth, augmenting the ability of ADWX-1 to occlude the pore of K_V_1.3. The mutation D33H reduced strong electrostatic repulsion between the neighboring AA, Asp^33^, and a conserved aspartic acid residue in the S-6 linker domain of K_V_1.3. Step 2: The polar interaction between residues in the toxin and channel were strengthened. The key residue that occludes the pore of K_V_1.3 is Lys^26^, therefore the neighboring hydrophobic AA, Ile^28^, was replaced with a polar threonine residue which resulted in a hydrogen bond between Thr^28^ (ADWX-1) and Asp^402^ (K_V_1.3) creating a more suitable docking environment. Step 3: A positively charged Arginine residue was introduced in the beginning of the α-helix domain (G11R) in order to create a salt bridge between four negatively charged residues in the upper most portion of K_V_1.3 with the critical residue identified as Asp^386^ [195].

Although challenges remain in improving selectivity and potency of potential peptide-toxin therapeutics, this work illustrates the utility of peptide-channel complex modeling in the development of diagnostic and therapeutic agents from existing toxin scaffolds.

#### 14.2.3. Mokatoxin-1

Using a scaffold-based/target-biased strategy developed by Takacs *et al*. (2009), a phage display was used to design and identify a synthetic peptide, Mokatoxin-1 (moka1), that is highly specific for K_V_1.3 [72]. Block of K_V_1.3 in T-cells counters the effects of anti-CD3/28 stimulation and suppresses effector cytokine secretion without cross-reactive gastrointestinal hyperactivity (side effects). Other scorpion toxins have been isolated which block K_V_1.3 (*i.e.*, KTX), however due to lack of channel specificity (KTX also blocks K_V_1.1 and K_V_1.2), undesirable side effects are produced (*i.e.*, diarrhea). 

A phage display library of 11,200 *de novo* proteins (as well as 20 of the original α-KTx peptides) was produced based on the α-KTx family scaffold (comprising 31 toxins; KTX being the primary template) and sorted using a high throughput selection strategy [72]. Three criteria were identified as critical in order to successfully utilize this technique, in addition to conservation of cysteine framework, bioengineered toxin variants must: (i) synthesize and fold correctly (regardless of phage anchorage); (ii) express topically on the phage surface so that toxins are accessible to the target (by genetic linkage to phage particle coat protein pIII); and (iii) successfully bind target channel despite phage cargo. 

The peptides produced displayed three major structural domains dubbed A (AAs 1–12), B (AAs 15–26), and C (AAs 30–38). Bioengineered Mokatoxin-1 has four positive charges at neutral pH, and is effectively chimeric with its domains corresponding to A (Ce3 toxin from *Centruroides elegans*), B (is present in AgTx2 & Agitoxin-3 (*Buthus occitanus*; AgTx3), and C (is present in both ChTx and Lq2 from the venom of *Leiurus quinquestriatus*). Once identified and structurally defined, Mokatoxin-1 was synthesized by Boc-SPPS and confirmed pharmacologically to be highly selective for K_V_1.3 [72]. Selectivity was based primarily on several key residues (a combination of residues not seen in any of the parent toxins): Leu^7^ and Pro^8^ (Ce3), Phe^22^ (AgTx2), and Arg^31^ and Tyr^33^ (ChTx), making Mokatoxin-1 an extremely successful example of rational drug design, and a template for further bioengineering of KCN scorpion toxins for the development of novel biopharmaceuticals.

Current methods to identify and develop molecular therapeutics based on KCN scorpion toxins have been retarded because isolating crude venom, shotgun venom gland sequencing, and site-directed mutagenesis are slow, and have thus yielded little in terms of tangible results. Because of this, efforts to rationally improve toxin-receptor selectivity are being explored.

### 14.3. Malaria

Annually there are upwards of 300 million new diagnoses of Malaria worldwide, one million of which can be fatal [197]. Artemisinin-based combination therapies are currently the standard treatment. Recently however, increasing resistance to existing pharmaceuticals has exacerbated the need for innovative antimalarial agents and treatment strategies.

A novel, low affinity KCN blocker named MeuTXKβ1 (*Mesobuthus eupeus*) has been isolated which produces unique cytolytic effects [198]. This activity is diverse, including inhibition of the parasite *Plasmodium berghei *(a gametocyte producer strain), binding of KCNs on rat brain synaptosomes and lysing of bacterium and various eukaryotic cells (oocytes and erythrocytes). Amazingly, a truncated *N*-terminus analog, N(1–21), displays anti-plasmodium activity without inducing haemolysis. This unique characteristic qualifies the peptide as an ideal template for the treatment of malaria, and opens the door for the development of transgenic malaria-resistant mosquitoes. Advanced bioengineering including cyclization, could potentiate the serum stability and half life of MeuTXKβ1-N(1–21), furthering its distinctive qualifications as a valuable molecular therapeutic. A second antimalarial scorpion toxin, meucin-24, has been identified from the genome (cDNA) of *Mesobuthus eupeus * [199]*. * Although the biological target of meucin-24 has not been confirmed, it shares high sequence identity with the *N*-terminus of a family of long chain KCN toxins (LcKTx), and represents an additional candidate for the development of antimalarial strategies.

## 15. Conclusions

As illustrated, scorpion toxins specific for potassium (K^+^) channels have been vital to an enormous amount of research resulting in the identification, localization and classification of novel channel types and families, as well as the creation of structural models pertaining to gating and binding of numerous ligands. The development of radiolabeled and fluorescent probes has enhanced our understanding of receptor pharmacology and membrane biophysics: information which has culminated in the development of pathological models involving KCN isoforms. 

The exhaustive use of combinatorial libraries by pharmaceutical industries, and the historical role of naturally derived molecules in the clinic, has amplified interest in biologics in recent years. Stimulated by our understanding of the importance of potassium channels in human physiology, improved technology and innovative techniques including cyclization and chimeric assembly, has expanded the role of toxins in medical research. 

As this work progresses, superior bioengineering techniques are continually being developed as evidenced by the use of phage epitope randomization and the scaffold-based/target-biased strategy in the design of novel molecules. The sophisticated application of these peptides will undoubtedly lead to a dramatic advance in the application of KCN scorpion toxins in molecular medicine.

## Appendix

### Structural Databases

Structural databases exist for many families of animal toxins [200,201] including Arachnoserver for spider toxins [202,203], and Conoserver for cone snail toxins [204,205,206]. At one point in time, a comprehensive database also existed for Scorpion toxins [207,208], unfortunately it has since become inactive. It is imperative that these informational bio-caches are maintained, and that regular updating persists. Utilized correctly, the structural information contained within could aid in the discovery and identification of novel compounds, and assist in the bioengineering of therapeutic scaffolds.
